# Combination of phospholipase Cε knockdown with GANT61 sensitizes castration-resistant prostate cancer cells to enzalutamide by suppressing the androgen receptor signaling pathway

**DOI:** 10.3892/or.2022.8426

**Published:** 2022-10-17

**Authors:** Wei Sun, Luo Li, Zhongbo Du, Zhen Quan, Mengjuan Yuan, Honglin Cheng, Yingying Gao, Chunli Luo, Xiaohou Wu

Oncol Rep 41: 2689–2702, 2019; DOI: 10.3892/or.2019.7054

Subsequently to the publication of this paper, an interested reader drew to the authors' attention that the same control β-actin bands had apparently been included in the western blots featured in Fig. 5E and F, even though different experiments were presented in these figure parts.

The authors have re-examined their data and realized that Fig. 5G was assembled incorrectly. The results from all the originally performed experiments were presented to the Editorial Office for our perusal. The revised version of Fig. 5, containing the correct β-actin data for the western blots in Fig. 5F, is shown on the next page. The authors regret the inadvertent error that was made during the preparation of Fig. 5, and confirm that this error did not seriously affect the conclusions reported in the paper. The authors are grateful to the Editor of *Oncology Reports* for allowing them the opportunity to publish a Corrigendum, and all the authors agree to this Corrigendum. Furthermore, they apologise to the readership for any inconvenience caused.

## Figures and Tables

**Figure 5.**
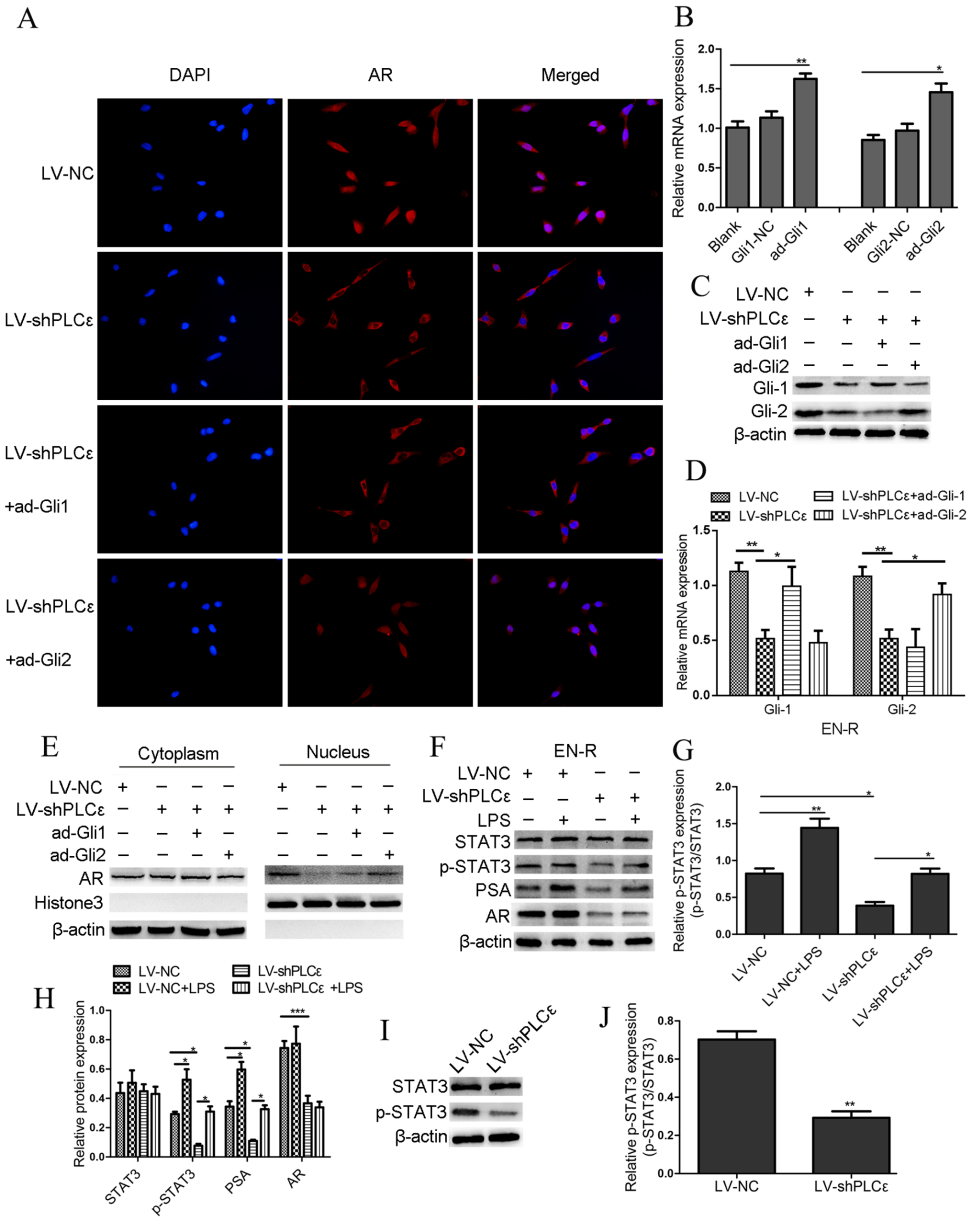
PLCε knockdown suppresses AR expression and nuclear translocation via different signaling pathways. (A) Immunofluorescence demonstrated AR intracellular distribution at 48 h following infection with LV-shPLCε and ad-Gli1/ad-Gli2 in EN-R cells. Magnification, ×400. PLCε knockdown inhibited AR nuclear translocation in EN-R cells. However, the overexpression of Gli-2 reversed the inhibitory effect produced by PLCε. (B) The relative mRNA expression level of Gli-1 and Gli-2 following treatment with an overexpression plasmid of ad-Gli1, ad-Gli2 was examined by RT-qPCR and β-actin served as loading control (NC stands for empty vector plasmid group (*P<0.05, **P<0.01). (C) Protein expression level of Gli-1 and Gli-2 following treatment with the overexpression plasmid of ad-Gli1, ad-Gli2 and PLCε knockdown. (D) The mRNA expression level of Gli-1 and Gli-2 following treatment with overexpression plasmid of ad-Gli1, ad-Gli2 and PLCε knockdown (*P<0.05, **P<0.01). (E) Western blotting showed that PLCε knockdown significantly decreased the AR expression in the nucleus. However, the expression of AR in the nucleus increased following Gli-2 overexpression. (F-H) Western blotting indicated that PLCε knockdown inhibited the PSA expression via the p-STAT3 signaling pathway (The results are represented as the mean ± SD; *P<0.05, **P<0.01 and ***P<0.001). (I and J) Western blotting showed that the downregulation of PLCε decreased the p-STAT3 protein expression in EN-R cells (The results are represented as the mean ± SD; **P<0.01). PLCε, phospholipase Cε; AR, androgen receptor; Gli, glioma-associated homolog; EN-R, enzalutamide-resistant cell line.

